# Glucose-regulated protein 78 modulates cell growth, epithelial–mesenchymal transition, and oxidative stress in the hyperplastic prostate

**DOI:** 10.1038/s41419-022-04522-4

**Published:** 2022-01-24

**Authors:** Xun Fu, Jianmin Liu, Daoquan Liu, Yongying Zhou, Yuhang Guo, Zhen Wang, Shu Yang, Weixiang He, Ping Chen, Xinghuan Wang, Michael E. DiSanto, Xinhua Zhang

**Affiliations:** 1 Department of Urology, Zhongnan Hospital of Wuhan University, Wuhan, China; 2 Department of Surgery and Biomedical Sciences, Cooper Medical School of Rowan University, Camden, NJ USA

**Keywords:** Prostatic diseases, Preclinical research

## Abstract

Benign prostatic hyperplasia (BPH) is a chronic condition which mainly affects elderly males. Existing scientific evidences have not completely revealed the pathogenesis of BPH. Glucose-regulated protein 78 (GRP78) is a member of the heat shock protein 70 superfamily, which serves as an important regulator in many diseases. This study aims at elucidating the role of GRP78 in the BPH process. Human prostate tissues, cultured human prostate cell lines (BPH-1 and WPMY-1) and clinical data from BPH patients were utilized. The expression and localization of GRP78 were determined with quantitative real time PCR (qRT-PCR), Western blotting and immunofluorescence staining. GRP78 knockdown and overexpression cell models were created with GRP78 siRNA and GRP78 plasmid transfection. With these models, cell viability, apoptosis rate, as well as marker levels for epithelial-mesenchymal transition (EMT) and oxidative stress (OS) were detected by CCK8 assay, flow cytometry analysis and Western blotting respectively. AKT/mTOR and MAPK/ERK pathways were also evaluated. Results showed GRP78 was localized in the epithelium and stroma of the prostate, with higher expression in BPH tissues. There was no significant difference in GRP78 expression between BPH-1 and WPMY-1 cell lines. In addition, GRP78 knockdown (KD) slowed cell growth and induced apoptosis, without effects on the cell cycle stage of both cell lines. Lack of GRP78 affected expression levels of markers for EMT and OS. Consistently, overexpression of GRP78 completely reversed all effects of knocking down GRP78. We further found that GRP78 modulated cell growth and OS via AKT/mTOR signaling, rather than the MAPK/ERK pathway. Overall, our novel data demonstrates that GRP78 plays a significant role in the development of BPH and suggests that GRP78 might be rediscovered as a new target for treatment of BPH.

## Introduction

Benign prostatic hyperplasia (BPH) is a common chronic disease in elderly men [1]. Accumulating evidence shows that its incidence increases with age [2]. More than 80% of men over 70 years old are troubled by this disease worldwide [3]. A number of pathophysiological hypotheses of BPH have been proposed, including the imbalance of androgen-estrogen ratio; the dysregulation of cell proliferation and apoptosis; the interaction between stromal and epithelial cells; inflammation; as well as growth factors [4]. Also, oxidative stress (OS) has been found associated with BPH over the past decades. The prostate cells are often subjected to severe OS due to obesity [5], smoking and aging [6, 7]. In addition, attention has been focused on epithelial-mesenchymal transition (EMT), a process implicated in embryonic development, organ fibrosis, cancer invasion and metastasis [8]. It is already well accepted that this process contributes to accumulation of stromal cells in the prostate [9]. However, the exact molecular mechanism of BPH has not been fully elucidated.

Glucose-regulated protein 78 (GRP78), also known as heat shock protein family A member 5, belongs to the HSP70 superfamily [10]. It mainly resides in the endoplasmic reticulum (ER) and functions as a molecular chaperone to start unfolded protein response (UPR) signaling during ER stress [11], which regulates intracellular Ca^2+^ homeostasis and insulin/IGF-1 signal transduction [12]. This molecule can also be found on the cell surface [13]. Under stressed conditions, GRP78 transduces to the cell membrane from the cytoplasm, leading to its abundant expression on the membrane [14]. A number of studies provide evidence for the vital role GRP78 plays in cell proliferation and apoptosis in a wide range of cells and tissues [15–19]. GRP78 is able to act as the target of steroids to modulate cell proliferation in hormone-sensitive tissues [19]. It is also essential for embryonic development and senility [12]. Actually, high level expression of GRP78 was observed at the blastocyst stage of mouse embryo [20]. Meanwhile, it was shown that GRP78 is highly expressed in the follicle, oviduct, embryo and placenta [21]. In addition to cell growth and embryonic development, the EMT has been implicated in the dysregulation of GRP78 [22], where it functions as a key regulator modulating several cell adhesion markers, including E-cadherin, N-cadherin, and Snail2 (Slug) [23, 24]. Also, GRP78 has been correlated with OS. In chronic kidney disease, GRP78 was overexpressed to counteract OS damage and produce an important protective effect [25]. In vascular endothelial cells, copper oxide nanoparticles (CuONPs)-induced OS leads to upregulation of GRP78 [26].

A link between GRP78 and BPH has not been reported in the literature. As mentioned above, BPH mainly results from the imbalance of cell proliferation and cell apoptosis with the proliferation being dominant. The occurrence of EMT, along with accumulation of reactive oxygen species (ROS), also have been demonstrated to contribute to BPH development [9, 27]. Recently, it was hypothesized that BPH implicated “reawakening” of the embryonic process where the prostate mesenchyme induced epithelial differentiation [28]. Based on all of the above findings, we speculated that GRP78 is associated with BPH. Indeed, our current study has revealed an upregulated expression of GRP78 in BPH tissues compared with normal ones. We further examined cell viability, cell apoptosis, as well as the status of EMT and OS after knockdown and overexpression of GRP78 in human prostate cell lines in order to investigate the effect of GRP78 on BPH development. Finally, we tried to identify the underlying molecular mechanism(s) through which GRP78 modulates proliferation, apoptosis and OS in BPH.

## Results

### The expression and localization of GRP78 in human BPH tissues and prostate cells

**Fig. 1:**
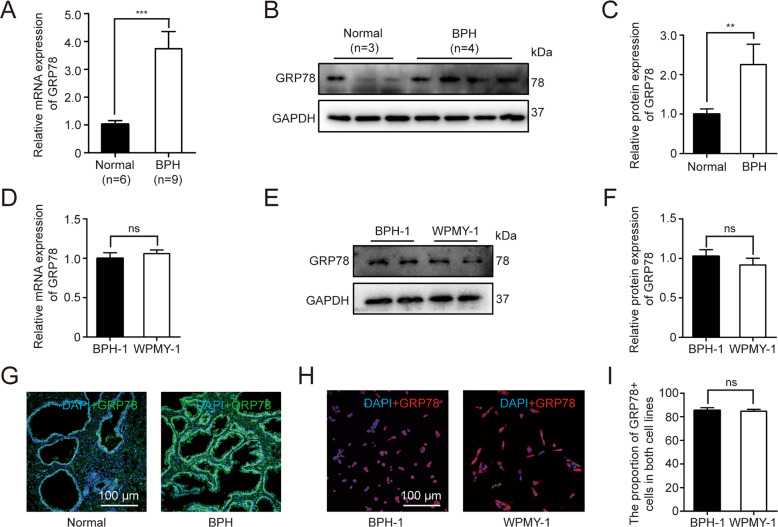
GRP78 is upregulated in hyperplasic prostate tissues and localized at stromal and epithelial regions of human prostate tissues. **A** The expression of GRP78 in BPH tissues vs normal tissues at the mRNA levels determined by qPCR. **B**, **C** Western blotting analysis and relative densitometric quantification of GRP78 in prostate tissues. **D** The mRNA expression of GRP78 in BPH-1 cells vs WPMY-1 cells. **E**, **F** The protein expression and relative densitometric quantification of GRP78 in two cell lines. **G** Immunofluorescence staining for GRP78 in the hyperplastic prostate and normal prostate. DAPI (blue) indicates the nucleus staining. Cy3-immunofluorescence (green) represents GRP78 protein staining. **H**, **I** Immunofluorescence staining for GRP78 in prostate cells. DAPI (blue) indicates the nucleus staining. Cy3-immunofluorescence (red) represents GRP78 protein staining. The proportion of GRP78 + cells was quantified. The scale bars are 100 μm. GAPDH is used as loading control. ns: *p* > 0.05; **p < 0.01; ***p < 0.001.

As shown in Fig. 1, GRP78 mRNA and protein levels were substantially upregulated in BPH tissues (Fig. 1A–C). There was no difference of GRP78 expression between BPH-1 and WPMY-1 cell lines (Fig. 1D–F). The fluorescence intensity of BPH samples was significantly higher than that of normal samples (Fig. 1G). In addition, GRP78 was localized in both epithelial and stromal compartments of prostate tissues (Fig. 1G), and more specifically, it was stained at the membrane or the cytoplasm, rather than the nucleus, of BPH-1 and WPMY-1 cell lines (Fig. 1H). The proportion of GRP78-stained cells also showed no difference between two lines in the immunofluorescence staining experiment (Fig. 1I).

### Knockdown of GRP78 attenuates cell viability and induces apoptosis in human prostate cells

GRP78-silenced cell models were established through GRP78 siRNA (siGRP78) transfection. The knockdown efficiency of si-1, si-2 and si-3 was assessed by qPCR, Western blotting and flow cytometry analysis. si-2 was shown to more strongly decrease GRP78 mRNA and protein levels in BPH-1 and WPMY-1 cells, compared with si-1 and si-3 (Fig. S1A–C). Moreover, si-2-mediated GRP78 knockdown induced apoptosis more effectively in prostate cell lines than the other two siRNAs (Fig. S1D, E). Therefore, si-2 was ultimately selected for our following experiments.

**Fig. 2:**
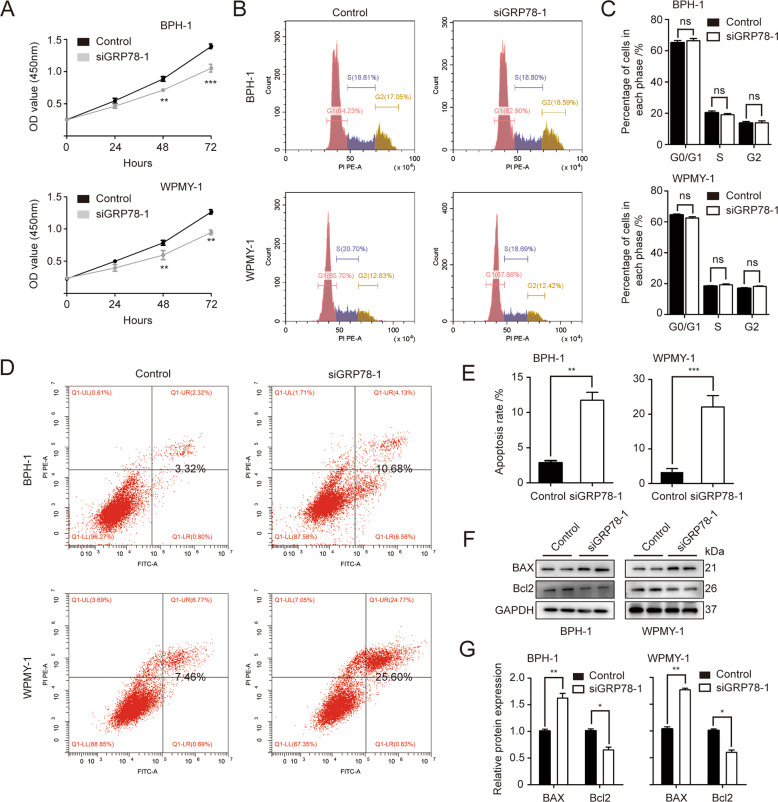
GRP78 knockdown inhibits proliferation and induces apoptosis of prostate cells. **A** Cell viability (OD value (450 mm)) of BPH-1 and WPMY-1 cells after GRP78 KD at different time points (0, 24, 48, 72 h) by CCK8 assay. **B** Cell cycle detection for BPH-1 and WPMY-1 by flow cytometry analysis. The percentages (%) of cell populations at different cell cycle stages are listed within the panels **C** Statistical analysis for the cell percentage in each cell cycle phase. **D** Flow cytometry analysis of prostate cell apoptosis after transfected with either control siRNA (Control, left) or siGRP78–1 (right). PI PE-A in y-axis stands for the fluorescence intensity of propidine iodide (PI), and FITC-A in x-axis stands for the fluorescence intensity of Fluorescein isothiocyanate (FITC) labeled Annexin V. The apoptosis rate is represented by percentage of Annexin V + /PI + cells. **E** Statistical analysis for apoptotic rate (%) of both prostate cell lines. **F**, **G** Immunoblot assay and relative densitometric quantification of apoptosis-related proteins BAX and Bcl2 in GRP78-silenced prostate cells. GAPDH is used as loading control. ns: *p* > 0.05; *p < 0.05; **p < 0.01; ***p < 0.001.

The effect of GRP78 silencing with si-2 (siGRP78–1) on cell proliferation, cell cycle and cell apoptosis was subsequently determined by CCK8 assay and flow cytometry analysis. GRP78 KD strongly inhibited cell viability at 48 h and 72 h in both BPH-1 and WPMY-1 cells (Fig. 2A). However, no statistically significant difference was observed in cell cycle detection (Fig. 2B, C). GRP78 silencing also greatly increased the apoptosis rate of two prostate cell lines by ~ 3.2 fold and 3.4 fold, respectively (Fig. 2D, E). In agreement with previous studies, BAX (a pro-apoptotic protein) was shown to downregulate while Bcl2 (an anti-apoptotic protein) was upregulated in BPH tissues (Fig. S2A, B). Consistently, BAX tended to increase while Bcl-2 was decreased in GRP78-silenced prostate cells (Fig. 2F, G).

### Overexpression of GRP78 promotes cell growth and inhibits apoptosis in human prostate cells

**Fig. 3:**
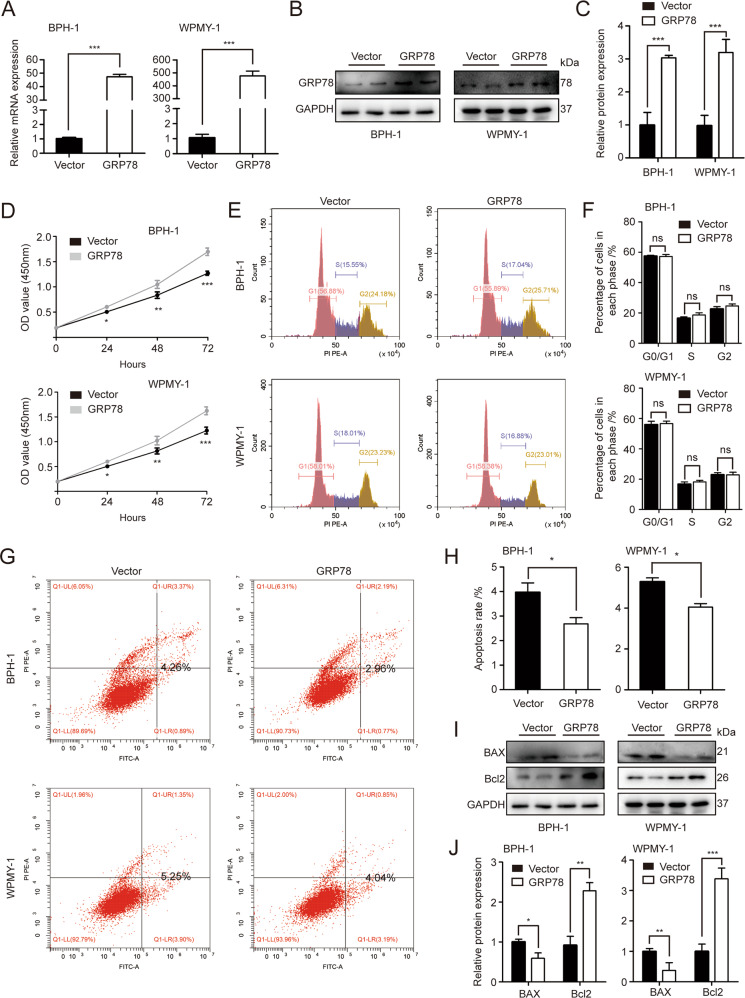
Overexpression of GRP78 suppresses apoptosis and enhances viability of BPH-1 and WPMY-1 cells. **A**, **B** GRP78 mRNA and protein levels in prostate cells transfected with either GRP78 plasmid or vector. **C** Relative densitometric quantification of GRP78 protein expression. **D** The viability of prostate cells after transfection at different time points (0, 24, 48, 72 h). **E** Cell cycle detection for BPH-1 and WPMY-1 by flow cytometry analysis. The percentages (%) of cell populations at different cell cycle stages are listed within the panels. **F** Statistical analysis for the cell percentage in each cell cycle phase. **G**, **H** Detection of prostate cell apoptosis by flow cytometry analysis. Apoptotic cells are stained with Annexin V and PI. The apoptosis rates were statistically analyzed. **I**, **J** The protein expression and relative densitometric quantification of BAX and Bcl2 in BPH-1 and WPMY-1 cells after transfection. GAPDH is used as loading control. *p < 0.05; **p < 0.01; ***p < 0.001.

We transfected GRP78 plasmid into BPH-1 and WPMY-1 cell lines. As shown in Fig. 3A–C, the plasmid substantially increased GRP78 transcript and translation levels in prostate cells. The viability of GRP78-overexpressed cells was significantly higher at 24, 48 and 72 h (Fig. 3D). In agreement with the results of gene KD, there was no difference for cell cycle stages between cells treated with vector and GRP78 plasmid (Fig. 3E, F). Furthermore, GRP78 overexpression significantly inhibited apoptosis of BPH-1 and WPMY-1 cells, as demonstrated by dramatically decreased apoptosis rates in Fig. 3G, H. Also, the protein expression of BAX was significantly repressed and Bcl-2 levels were enhanced (Fig. 3I, J).

### GRP78 participates in activation of AKT-mTOR signal transduction pathway

**Fig. 4:**
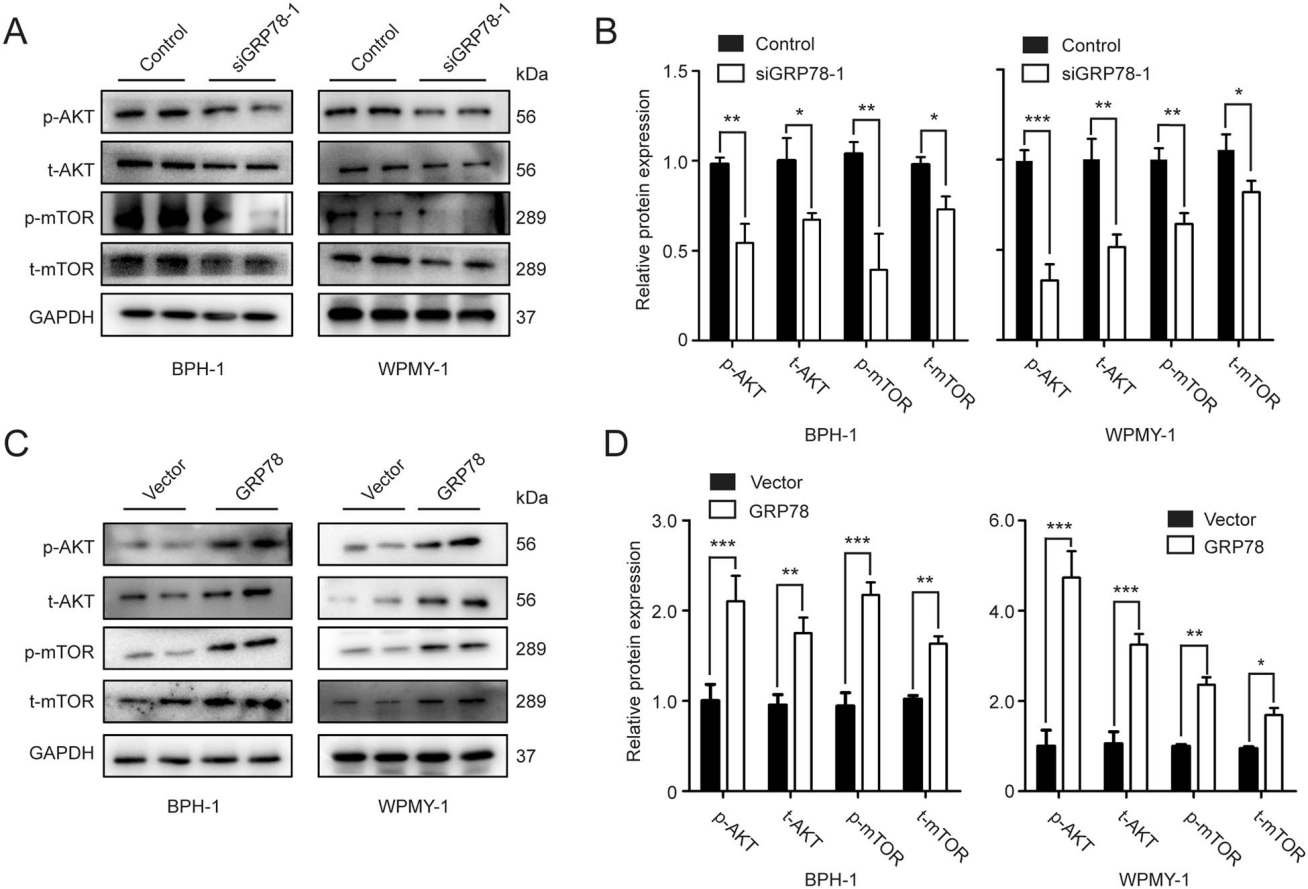
GRP78 modulates apoptosis-related AKT/mTOR signaling in prostate cells. **A**, **B** Immunoblot assay and relative densitometric quantification of four molecular markers for AKT signal pathway, t-AKT, p-AKT, t-mTOR and p-mTOR, in GRP78-silenced prostate cells. **C**, **D** Immunoblot assay and relative densitometric quantification of four molecular markers for AKT signal pathway in GRP78-overexpressed cells. GAPDH is used as loading control. *p < 0.05; **p < 0.01; ***p < 0.001.

The signaling pathways correlating with GRP78-related cell growth and death were investigated in this section. The protein expression levels of four AKT-mTOR signaling biomarkers, p-AKT, t-AKT, p-mTOR and t-mTOR, were detected in GRP78-silenced and GRP78-overexpressed cells. Figure 4A, B show a significant downregulation in protein expression of these biomarkers in GRP78 KD cells (BPH-1 and WPMY-1). Meanwhile, the ratio of p-AKT/t-AKT and p-mTOR/t-mTOR also decreased (Fig. 4A, B). In contrast, GRP78 overexpression dramatically elevated levels of four AKT/mTOR signaling markers, as well as the ratio of p-AKT/t-AKT and p-mTOR/t-mTOR (Fig. 4C, D). However, GRP78 silencing did not significantly change protein levels of several MAPK signal pathway biomarkers, including ERK1/2, p38 and JNK (Fig. S3A–D).

### SC79 reverses the effect of GRP78 knockdown on prostate cells

**Fig. 5:**
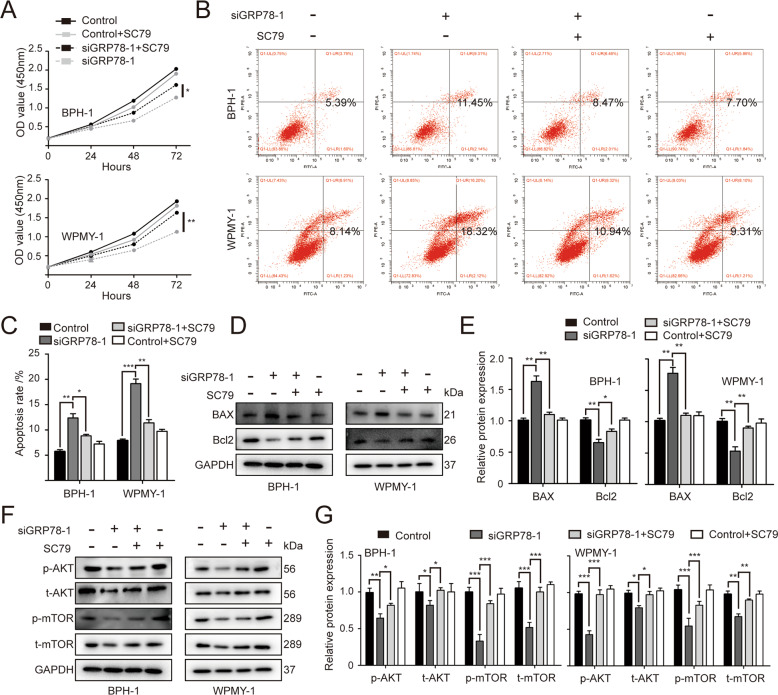
SC79 recovers the effect of GRP78 knockdown on prostate cells. **A** The viability of BPH-1 and WPMY-1 cells detected by CCK8 assay at different time point (0, 24, 48, 72 h). OD value (450 mm) is considered as cell viability. **B**, **C** Flow cytometry analysis for apoptosis of BPH-1 and WPMY-1 cells. Both cell lines were transfected with either siControl or siGRP78–1, and then treated with/without SC79 at 4 μg/ml. The apoptosis rates were statistically analyzed. **D**, **E** Western blotting and relative densitometric quantification for apoptosis–related protein (BAX and Bcl2) expression in prostate cells. **F**, **G** Immunoblot assay and relative densitometric quantification for four molecular targets of AKT/mTOR pathway. GAPDH is used as loading control. *p < 0.05; **p < 0.01; ***p < 0.001.

To further validate whether siGRP78-mediated apoptosis implicated AKT-mTOR signal pathway, AKT activator SC79 was used to treat BPH-1 and WPMY-1 cells transfected with/without GRP78 siRNA. As expected, SC79 completely reversed attenuated cell viability caused by GRP78 silencing (Fig. 5A). siGRP78-induced apoptosis was rescued by SC79 (Fig. 5B, C). Furthermore, this activator recovered the expression of apoptosis-related proteins and AKT/mTOR markers changed by GRP78 knockdown in BPH-1 and WPMY-1 lines (Fig. 5D–G).

### GRP78 modulates the EMT process in BPH-1 cells

**Fig. 6:**
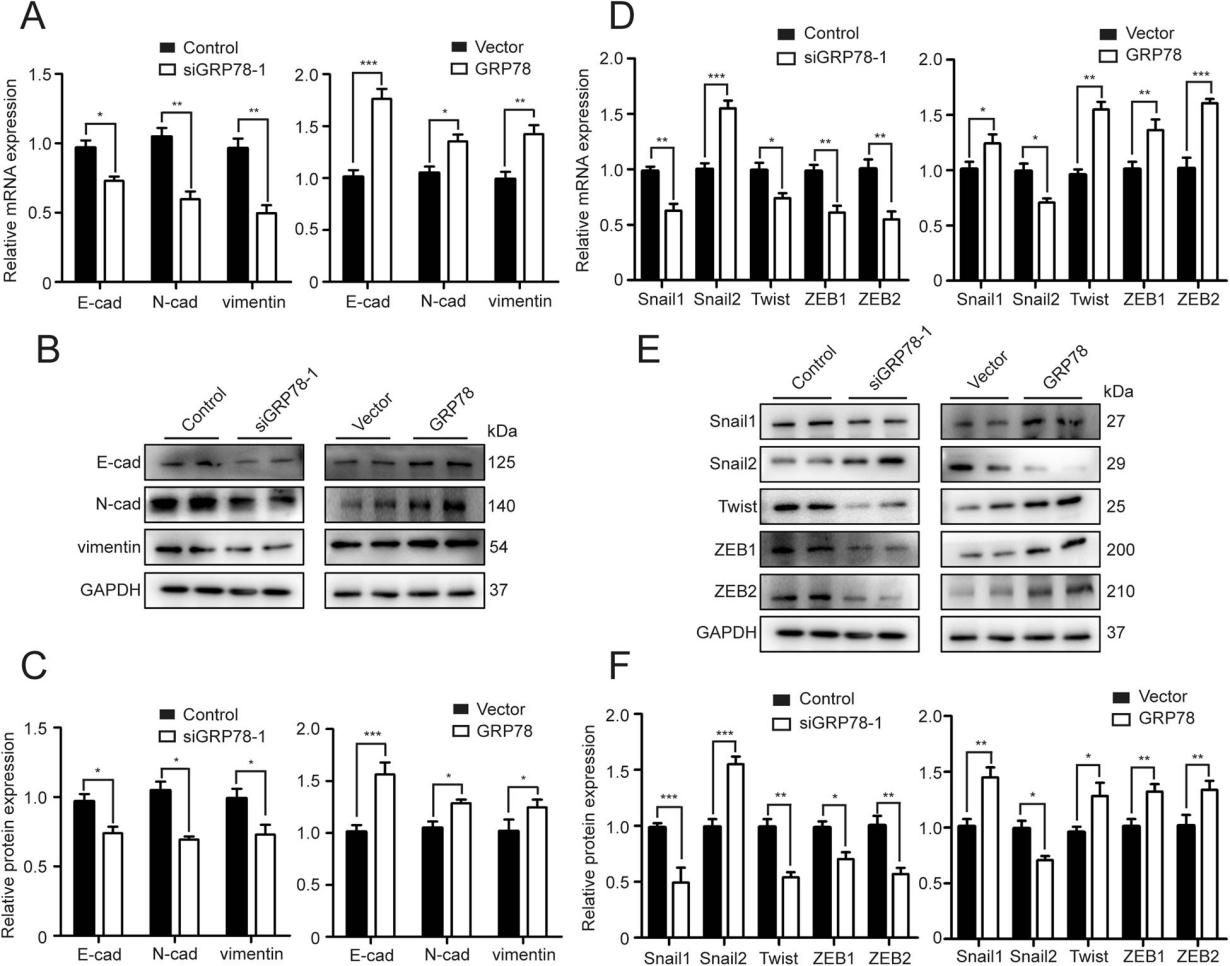
GRP78 regulates multiple EMT markers in BPH-1 cells. **A**, **B** The mRNA and protein expression of E-cad, N-cad and vimentin in GRP78-silenced or GRP78-overexpressed BPH-1 cells. **C** Relative densitometric quantification of three EMT markers. **D**, **E** The mRNA and protein expression of several EMT-TFs (Snail1, Snail2, Twist, ZEB1 and ZEB2) in BPH-1 cells. **F** Relative densitometric quantification of these EMT-TFs. In all figures, GAPDH is used as loading control. *p < 0.05; **p < 0.01; ***p < 0.001.

To investigate whether GRP78 participated in the EMT process, we determined the expression of EMT markers (E-cad, N-cad and vimentin) as well as EMT transcript factors (EMT-TFs) (Snail1, Snail2, Twist, ZEB1 and ZEB2) in BPH-1 cells. As shown in Fig. 6A–C, GRP78 knockdown dramatically downregulated E-cad, N-cad and vimentin, while GRP78 overexpression significantly increased expression of these markers. GRP78-silenced cells displayed a substantial reduction of Snail1, Twist, ZEB1 and ZEB2, as well as an upregulation of Snail2 at both mRNA and protein levels, while GRP78 overexpression reversed all these changes (Fig. 6D–F).

### GRP78 regulates oxidative stress via AKT activation in prostate cells

**Fig. 7:**
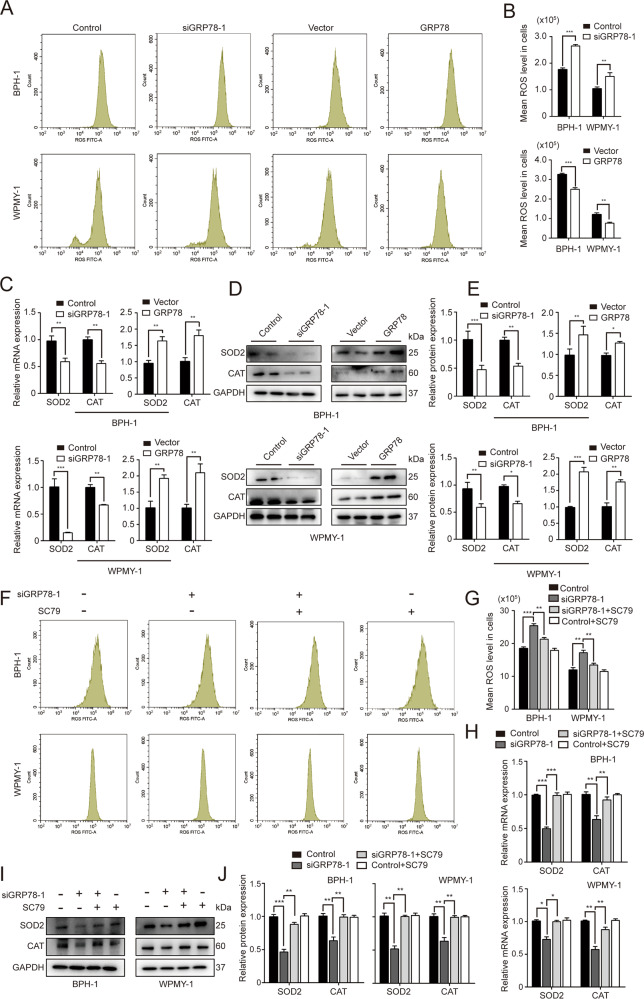
GRP78 modulates ROS production and expression of antioxidant enzymes in prostate cells. **A** Flow cytometry analysis for ROS generation in BPH-1 and WPMY-1 cells with either GRP78 knockdown or GRP78 overexpression. **B** The statistical analysis for mean ROS level. **C** The mRNA levels of SOD2 and CAT in both prostate cell lines after transfection by qPCR. **D**, **E** Immunoblot assay and relative densitometric quantification for SOD2 and CAT in prostate cells. **F**, **G** Flow cytometry analysis for ROS generation of BPH-1 and WPMY-1 cells. Two lines were transfected with siRNA and treated with/without SC79. The mean ROS level was statistically analyzed. **H**, **I** The mRNA and protein levels of SOD2 and CAT in two lines treated with siRNA and with/without SC79. **J** Relative densitometric quantification of SOD2 and CAT. GAPDH is used as loading control. *p < 0.05; **p < 0.01; ***p < 0.001.

We further measured ROS production and expression of two antioxidant enzymes: superoxide dismutase-2 (SOD2) and catalase (CAT) in BPH-1 and WPMY-1 cells. As shown in Fig. 7A, B and Table S1, GRP78 KD resulted in excessive levels of ROS. In contrast, ROS levels were significantly lower in GRP78-overexpressed prostate cells. Knockdown of GRP78 dramatically decreased mRNA and protein levels of SOD2 and CAT, while overexpression of GRP78 significantly upregulated these two genes (Fig. 7C–E). Additionally, we investigated whether AKT participated in these changes related to GRP78. As expected, AKT activator SC79 rescued ROS increase caused by GRP78 silencing (Fig. 7F, G and Table S2). The mRNA and protein expression of SOD2 and CAT downregulated by GRP78 knockdown were also recovered by SC79 (Fig. 7H–J).

### The modulation of cell growth, cell apoptosis, as well as EMT and OS status by GRP78 is further validated by another siRNA (siGRP78–2) and another cell line RWPE-1

**Fig. 8:**
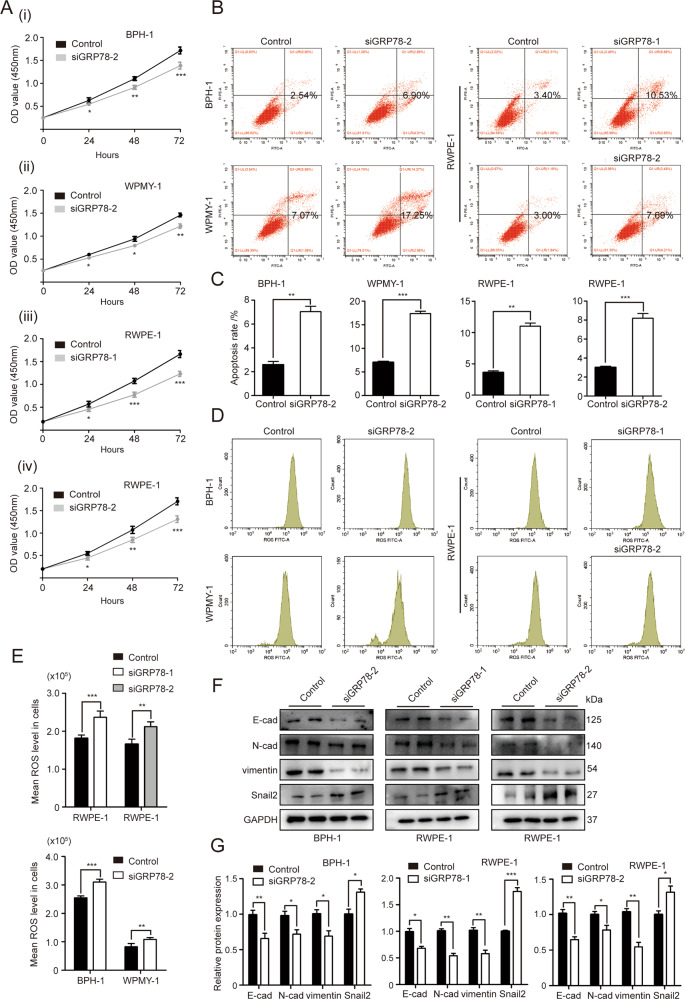
GRP78 regulates proliferation, apoptosis, as well as EMT and OS status in prostate cells validated by siGRP78–2 and another cell line RWPE-1. **A** Cell viability of BPH-1, WPMY-1 and RWPE-1 cells after GRP78 knockdown with siGRP78–1 or siGRP78–2 at different time points (0, 24, 48, 72 h) by CCK8 assay. **B** Flow cytometry analysis of prostate cell apoptosis after transfection with siGRP78. **C** Statistical analysis for apoptotic rate (%) of three prostate cell lines. **D** Flow cytometry analysis for ROS generation in three cell lines transfected with either siGRP78–1 or siGRP78–2. **E** The statistical analysis for mean ROS level. **F**, **G** Immunoblot assay and relative densitometric quantification of EMT markers (E-cad, N-cad, vimentin and Snail2) in GRP78-silenced BPH-1 cells. GAPDH is used as loading control. *p < 0.05; **p < 0.01; ***p < 0.001.

We used another siRNA (siGRP78–2) and another cell line (RWPE-1) to repeat our phenotype observations to make our study more convincing. As expected, cell viability was repressed by siGRP78–2 in BPH-1, WPMY-1 and RWPE-1 cells (Fig. 8A). A dramatic increase in the proportion of apoptotic cells following GRP78 KD by siGRP78–2 was observed in these three cell lines (Fig. 8B, C). Silencing GRP78 with both siGRP78–1 and siGRP78–2 was shown to increase ROS levels, and downregulate SOD2 and CAT expression in three lines (Fig. 8D, E). In agreement with above-mentioned results, both GRP78-silenced prostatic epithelial cells (BPH-1 and RWPE-1) displayed a downregulation of E-cad, N-cad and vimentin, concomitant with an upregulation of Snail2 expression (Fig. 8F, G).

### GRP78 correlates with the severity of nocturia and expression of multiple markers for apoptosis, EMT and OS

Finally, we investigated the correlation between GRP78 and multiple clinical characteristics, as well as several markers of apoptosis, EMT and OS (BAX, Bcl2, E-cad, N-cad, SOD2 and CAT), by Pearson correlation analysis. The severity of nocturia in BPH significantly correlated with GRP78 levels (Table S3, *p* < 0.01), but no significant correlation was observed between other clinical characteristics and GRP78 expression. In addition, we found that GRP78 was negatively correlated with BAX and positively correlated with another apoptosis marker (Bcl2), EMT markers (E-cad, N-cad) and OS markers (SOD2, CAT) (Table S4).

## Discussion

This study showed that GRP78 is upregulated in BPH tissues and almost equally localized in both the stroma and epithelium of the prostate. We demonstrated GRP78 modulates the proliferation and apoptosis of prostate cells via AKT/mTOR signaling in vitro. We also found that GRP78 plays a key role in EMT and OS, and that AKT participates in this GRP78-regulated OS process. In general, our novel data suggests that GRP78 is closely related to BPH.

GRP78, a member of HSP70 superfamily, is an important molecular chaperone in the ER [10, 11]. An analysis of the human tissue-specific expression by genome-wide integration of transcriptomics showed that GRP78 is broadly expressed in human thyroid (rpkm 442.6), bone marrow (rpkm 284.6) and 24 other tissues, including the prostate (rpkm 169.4) [29]. This molecule was also reported to upregulate in multiple malignancies, including lung cancer, prostate cancer and glioblastoma [10, 14, 30]. In addition to cancer, GRP78 participates in embryonic development and senility [12]. Nevertheless, its function in BPH has not been completely identified. Actually, BPH is regarded as “reawakening” of the embryonic process with aging being one of the main pathogenic factors [28]. We therefore speculated that GRP78 likely drove the development and progression of BPH. Indeed, we observed an upregulation of GRP78 in both epithelial and stromal compartments of hyperplastic prostate tissues. The GRP78 molecule was shown to be almost equally localized on the membrane or in the cytoplasm of human prostate cell lines, with relatively higher expression on the cell surface.

GRP78 was found as a key regulator of cell survival, proliferation and apoptosis in a wide range of tissues [12, 15, 21, 31]. Excessive levels of GRP78 augmented cartilage cell proliferation during the chondrogenesis process [32]. Knockdown of GRP78 slowed the cell growth rate and induced cell apoptosis in glioma [33]. Similarly, our current study revealed that the viability of BPH-1 and WPMY-1 cells is attenuated when GRP78 silenced. Meanwhile, siGRP78 caused a dramatically increased apoptosis rate as well. It is interesting to observe that the apoptosis rate of GRP78-silenced WPMY-1 cells was 25.60%, nearly two times higher than that of BPH-1 cells, which was probably attributed to the difference of cell viability after incubation with siRNA and transfection reagents. Besides cells in the GRP78 KD group, the apoptosis rate of WPMY-1 cells in the control group (transfected with scrambled siRNA and transfection reagents) was 7.46%, which was also approximately twice as much as that of BPH-1 cells (3.32%). These observations indicate that WPMY-1 is more sensitive to siRNA (control siRNA or GRP78 siRNA) and/or transfection reagents than BPH-1 cells. On the other hand, both cell lines showed similar growth multiples in apoptosis rate (~3.2 fold in BPH-1 and ~3.4 fold in WPMY-1), indicating that the effect of GRP78 KD on both lines is almost same, when taking into account the similar basal expression of GRP78 in BPH-1 and WPMY-1. Also, the CCK8 assay revealed a relatively poorer viability of WPMY-1 cells at the time point 72 h, compared with that of BPH-1 cells, following GRP78 KD. Thus, we speculated that it was the difference of cell sensitivity to siRNA and/or transfection reagents, rather than a different amount of GRP78 in two cell lines, that accounted for the different impact on the overall level of cell apoptosis. Additionally, apoptosis-related proteins, BAX (a pro-apoptotic molecule) and Bcl2 (an anti-apoptotic molecule), were significantly changed, both of which strictly regulates the intrinsic apoptotic pathway [34]. However, no significant differences in terms of cell cycles were observed in either BPH-1 or WPMY-1 cells. We inferred from these findings that the suppression of cell viability induced by GRP78 knockdown might not correlate with cell cycle arrest.

The current study also revealed that GRP78 knockdown results in the repression of the EMT process with decreased expression of epithelial phenotype marker E-cad, and mesenchymal phenotype markers (N-cad and vimentin). This was unexpected because EMT inhibition is normally compatible with upregulation of the epithelial marker, along with downregulation of mesenchymal markers [35, 36]. To investigate the exact mechanism by which E-cad decreased, we determined the expression of multiple EMT-TFs, including Snail1, Snail2, Twist, ZEB1 and ZEB2, and found that siGRP78 dramatically upregulated Snail2 while repressing the expression of other EMT-TFs. In fact, GRP78 KD has been shown to induce Snail2 expression and Snail2 was reported as a strong suppressor of E-cad in the prostate [23]. Therefore, the abnormal changes of E-cad expression is probably due to the upregulation of Snail2.

We also investigated the OS status in GRP78-silenced prostate cells. SOD2 and CAT, two critical antioxidant enzymes, are shown to be involved in the defense mechanism against OS and they are usually used to gage the antioxidant activity directly and the OS degree indirectly [37]. It is generally known that OS can not only activate the transcription factor NF-κB that targets at inflammation, cell proliferation, cell migration, and apoptosis, but also lead to DNA damage via vascular tissue damage, protein structure and function damage, genomic damage, through both of which compensatory cellular proliferation is induced and BPH ultimately occurs [27]. In contrast, our study reveals that OS is significantly inhibited by overexpression of GRP78 while it is activated with GRP78 knockdown. These findings seemed unexpected as both GRP78 and OS are stimuli for BPH. However, Bi *et al*. revealed that GRP78 is capable of inhibiting ROS accumulation by an activation of AKT (protein kinase B, PKB) [38]. GRP78 was also shown to prevent OS via regulation of Ca^2+^ homeostasis by Liu’s group [39]. Interestingly, our study shows a dramatic decrease of AKT in GRP78-silenced prostate cells. Therefore, it was speculated that GRP78 itself was possibly an inhibitor for ROS generation and OS activation by regulating Ca^2+^ levels. It was also likely that AKT repression participated in excessive ROS production induced by GRP78 silencing. We also validated all aforementioned phenotype observations with another siRNA (siGRP78–2) and another cell lines (RWPE-1). Thus, the chance off-target effects was minimized. Notably, these phenotypic changes produced by GRP78 knockdown were completely reversed by GRP78 overexpression.

We further investigated the underlying mechanism through which GRP78 regulates the proliferation, apoptosis and OS in prostate cells. Existing evidence suggests that several signaling pathways, including AKT/mammalian target of rapamycin (mTOR) (AKT/mTOR) signaling and mitogen-activated protein kinases (MAPK)/extracellular regulated protein kinases (ERK) (MAPK/ERK) pathway, are involved in the regulation of cell apoptosis [40, 41]. The MAPK signaling cascade includes three kinases: MAPK kinase kinase (MAPKKK), MAPK kinase (MAPKK) and MAPK, all of which transmit signals by phosphorylating downstream molecules [42]. Four different MAPK cascades have been reported to exist in cells, including ERK1/2, c-Jun N-terminal kinase (JNK), p38 MAPK and ERK5 [43], with MAPK/ERK cascades playing more important roles in regulating cell proliferation and cell apoptosis [42]. AKT/mTOR signaling is also a significant apoptosis-related signal transduction pathway which mainly affects cell survival, proliferation, apoptosis, cell cycle and actin remodeling [44]. Briefly, the activation of AKT upstream protein, phosphatidylinositol-3-kinase (PI3K), leads to the successive phosphorylation of multiple downstream molecules, from AKT ultimately to mTOR (mTORC1 and mTORC2) [44]. The phosphorylation of mTOR could trigger a series of the above-mentioned biological processes [44]. The apoptosis of osteoarthritic chondrocytes, human hepatocytes and keratinocytes has been reported to implicate these two pathways [45–47]. Meanwhile, Ni *et al*. found that GRP78 is capable of forming complexes with other protein partners to activate AKT/mTOR signaling [48]. In our current study, we showed that lack of GRP78 strongly represses the AKT/mTOR pathway while GRP78 overexpression activated this signaling. However, there were no statistically significant changes in protein expression of MAPK/ERK signaling markers in GRP78-silenced BPH-1 or WPMY-1 cells. We then combined SC79, an AKT activator which augments AKT phosphorylation through binding to PH domain of AKT, with siGRP78 to treat cultured human prostate cell lines. We showed the viability and apoptosis level of BPH-1 and WPMY-1 cells that were altered by siGRP78 were completely reversed to control levels. In agreement with our finding, Liu *et al*. showed the GRP78 blockade with a specific antibody (MAAb159) decreased PI3K instead of MAPK targets, indicating that GRP78 was the upstream regulator of AKT/mTOR signaling [49, 50]. We therefore speculated that it was the AKT/mTOR pathway, rather than MAPK/ERK pathway, that probably participated in regulating the cell growth related to GRP78. In addition to cell growth, AKT/mTOR signaling is also involved in GRP78-modulated OS status changes. SC79 could recover the excessive ROS production and decrease of SOD2 and CAT expression caused by GRP78 knockdown. This was compatible with the previous study mentioned above [38]. Thus, AKT indeed participates in OS status changes related to GRP78. This could also explain why GRP78 knockdown increased ROS levels and downregulated SOD2 and CAT though both GRP78 and OS are stimuli for BPH. Interestingly, all these phenotypes (cell viability, apoptosis and OS status) of control cell lines were not significantly affected by SC79 alone, and the phosphorylation of AKT was promoted insignificantly. This condition was consistent with a previous publication by Xu *et al*. [51], who also found an insignificant enhancement of the phosphorylation of AKT by SC79. We inferred that it was probably due to the fact that AKT phosphorylation had reached its maximum in our culture conditions since SC79 did not affect ROS production and expression of SOD2 and CAT in control lines. Only when the phosphorylation of AKT was repressed (silencing GRP78 in this study) would we observe a significant increase of p-AKT expression induced by SC79.

Finally, our current study shows that the severity of nocturia correlated with GRP78 expression levels in BPH patients, whereas there was no significant correlation between other clinical characteristics and GRP78 expression. In addition, GRP78 was shown to be negatively correlated with BAX and positively correlate with another apoptosis marker (Bcl2), EMT markers (E-cad, N-cad) as well as OS markers (SOD2, CAT) in BPH tissues, indicating a significantly positive correlation between GRP78 and OS status, EMT status, as well as apoptosis. These results were compatible with all of the above findings. The correlation between GRP78 and clinical traits, as well as these important biological processes, are worthy of further study.

In conclusion, we reveal and substantiate a correlation between GRP78 and BPH in this study. GRP78 was shown to have higher expression in the hyperplastic prostate and its upregulation mediates the proliferation and apoptosis of prostate cells via the AKT/mTOR signal pathway. Meanwhile, GRP78 plays a key role in EMT and OS, and it regulates OS status via AKT activation in BPH. In aggregate, our novel data provides evidence that GRP78 is probably a key regulator of BPH and it could likely be considered as a new therapeutic target for BPH.

## Materials and methods

### Human tissues and clinical data

Normal prostate samples were obtained from six brain-dead men (mean age: 29.6 ± 4.8 years old), with pathological examination by two different pathologists revealing no hyperplasia. A total of 104 BPH specimens (mean age, 70.1 ± 2.5 years old) with clinical data were obtained from the patients who underwent transurethral resection prostate in the department of urology, Zhongnan Hospital of Wuhan University. Post-operative pathological examination revealed BPH. Prostate tissues were divided into two strips and were, respectively, stored in liquid nitrogen for qPCR and Western blotting, and stored in 10% neutral buffered formalin for immunofluorescence microscopy and tissue microarray (TMA) construction. All prostate samples were collected with written informed consent from Zhongnan Hospital of Wuhan University and the collection was approved by the Hospital Medical Ethics Committees. Our human studies were conducted in accordance with the principles of the Declaration of Helsinki.

### Cell culture

Human BPH epithelial cell line (BPH-1) (Cat. #BNCC339850) was purchased from Procell Co., Ltd., Wuhan, China, and was cultured in RPMI-1640 medium (Gibco, China) with 10% fetal bovine serum (FBS, GIBCO, Australia). SV40 large T antigen immortalized stromal cell line (WPMY-1) (Cat. #GNHu36) was acquired from Stem Cell Bank, Chinese Academy of Sciences in Shanghai, China, and it was cultured in DMEM medium (Gibco, China) with 5% FBS. Human normal prostatic epithelial cell line (RWPE-1) (Cat. #CRL-11609) was obtained from American Type Culture Collection (ATCC). The cells were cultured in prostate epithelial cell medium (PEpiCM, ScienCell Research Laboratories) with 1% prostate epithelial cell growth supplement (PEpiCGS, ScienCell Research Laboratories) and 1% penicillin/streptomycin solution (P/S, ScienCell Research Laboratories). Identification of three cell lines was performed at the China Center for Type Culture Collection in Wuhan, China. All the cell lines were cultured at 37 °C, 5% CO_2_ conditions in the cell incubator.

### Cell transfection

The GRP78-target specific small interfering RNA (GRP78 siRNA, siGRP78) was purchased from Genepharma Ltd., Suzhou, China. Three different siRNAs (Sequences are listed in Table S5) were tested for highest GRP78 knockdown efficiency. As a control, negative control siRNA (Control) was used. GRP78 siRNAs (or control siRNA) were diluted in Opti-MEM reduced serum medium and mixed with Lipofectamine®2000 (Invitrogen, USA) according to the manufacturer’s instructions. The complexes were added to 6-well plates where cells were seeded in and cultured for at least 24 h. For GRP78 overexpression, GRP78 cDNA was synthesized and transduced into a plasmid by Fenghuishengwu Co., Ltd. in Changsha, China. Empty plasmid (vector) was used as control. After transfection by plasmid for 48 h, alterations of GRP78 at transcriptional and protein levels were evaluated by qRT-PCR and Western blotting.

### AKT activator treatment for rescue experiments

After siRNA transfection, BPH-1 and WPMY-1 cells were treated with AKT activator SC79 (MedChemExpress, China) at 4 ug/ml to activate AKT [52]. Cells in the untreated group were incubated with a corresponding amount of 0.1% DMSO. Both groups were subjected to RNA interference and then cell viability and apoptosis rate were measured by a cell counting kit-8 (CCK-8) assay and flow cytometry analysis/Western blotting, respectively.

### Cell Counting Kit-8 (CCK8) assay

Cell viability was examined by CCK-8 (MedChemExpress, China) assay in our study. Briefly, the neurons (approximately 5000 cells/well) were seeded in 96-well plate and subjected to various treatments described above. BPH-1 and WPMY-1 cells were then cultured in the cell incubator for 0, 24, 48 or 72 h, respectively. At different time points, 10 μl CCK-8 solution (Sangon Biotech, Shanghai, China) was added to each well, and cells were incubated in the dark for 1 h. The absorbance at 450 nm was measured by a microplate reader (Thermo Labsystems, Vantaa, Finland).

### Flow cytometry analysis

After transfection with siRNA or GRP78 plasmid, BPH-1 and WPMY-1 cells were harvested for flow cytometry analysis. The cell cycle stage was detected using the cell cycle staining kit (Multisciences Biotech, CO., Ltd. China) by flow cytometry (Beckman, Cat. #FC500). After phosphate-buffered saline (PBS) washing and centrifugation, the prostate cells were resuspended with 1 ml DNA staining solution and 10 μl permeabilization solution and then incubated in the dark at 37 °C for 30 min. For cell apoptosis detection, cells were resuspended with 1 ml pf binding buffer and stained with 5 μl Annexin V- FITC, as well as 10 μl propidium iodide (PI) (Multisciences Biotech Co., Ltd. China) for 5 min according to manufacturer’s instructions. To determine the level of ROS generation, cells were incubated with DCFH-DA at a 1:100 dilution in the dark for 30 min, followed by immediate detection of ROS levels by flow cytometry.

### Total RNA extraction, reverse transcription and quantitative real time PCR (qRT-PCR)

Total RNA was isolated from collected tissues and cells with RaPure Total RNA Micro Kit (Magen, China) and Trizol reagent (Invitrog, Carlsbad, CA, USA) according to the manufactures’ protocol. The RNA was quantified at 260 nm / 280 nm using the NanoPhotometer spectrophotometer (IMPLEN, Westlake Village, CA, USA). For reverse transcription, 2 μg of total RNA was reverse-transcribed into cDNA with ABScript II RT Master Mix (ABclonal, Wuhan, China) following manufacturer’s instructions. qRT-PCR was performed to determine the mRNA level of a gene of interest based on SYBR green in a Bio-Rad (Hercules, CA, USA) CFX96 system. The primer sequences are listed in Table S6. The relative mRNA expression levels of target genes to loading control GAPDH were calculated with 2^−ΔΔ*CT*^ method.

### Western blotting analysis

Tissues and harvested cells were lysed and ultrasonicated in RIPA reagent containing protease inhibitor and phosphatase inhibitor (Sigma-Aldrich) on ice for 30 min. The supernatant was collected after centrifugation at 14,000 × g for 10 min at 4 °C. The protein concentration was measured by bicinchoninic acid (BCA) assay. Protein extracts were resolved on 10% sodium dodecylsulfate-polyacrylamide (SDS-PAGE) gels (Wuhan Boster Biological Technology Ltd., Wuhan, China) prior to being transferred to polyvinylidene fluoride (PVDF) membrane (Millipore, Billerica, MA, USA). After blocking in 5% skim milk for 2 h, the membrane was incubated in primary antibody (Table S7) overnight at 4 °C and then incubated with secondary antibody: goat anti-rabbit IgG or goat anti-mouse IgG (Table S8) for 2 h at room temperature. Our bands were visualized with enhanced chemiluminescence kit (Thermo Scientific Fisher, Waltham, MA, USA) on a Tanon-5200 ECL imager (Tanon, Shanghai, China). The protein levels were quantified using Image J software. Target protein bands were normalized to the loading control GAPDH.

### Cell immunofluorescence staining

The prostate cells were grown on 12 mm coverslips in a 6-well plate and then washed with ice cold PBS. The coverslips were next fixed with 4% paraformaldehyde solution (PFA) for 30 min, followed by 0.1% Triton X-100 incubation at room temperature for 5 min and 10% BSA blocking at 37 °C for 1 h. Then, cells were successively incubated with primary antibody (Table S7) at 4 °C overnight, washed with PBS, and incubated with Cy3 labeled or FITC labeled secondary antibody (Table S8) in humidified air at 37 °C for 1 h. The nuclei were labeled blue with 2 μg/ml 4′,6-diamidino-2-phenylindole (DAPI). Visualization was performed with a Laser Scanning Confocal fluorescence microscope (cat. no. IX73; Olympus, Japan).

### Tissue immunofluorescence staining

Tissues were fixed with 4% PFA overnight at 4°C and embedded into paraffin (Paraplast, Sigma-Aldrich) using a special tissue processor (Thermo Fisher Scientific, Cat. #STP120). Paraffin sections (4 μm) were sliced using a rotary microtome (Thermo Fisher Scientific, Cat. #HM325). The slides were washed in PBS and incubated for 2 h in the mixture of PBS supplemented with 0.2% Triton X-100 and 0.1% bovine serum albumin, followed by incubation in humidified air with GRP78 primary antibody (Table S7) and Cy3-conjugated goat anti-rabbit IgG (Table S8) in turn. DAPI was used to stain the nucleus. A confocal fluorescence microscope was used to visualize each slide.

### TMA construction

The TMA was constructed based on the methods our group previously reported [53]. Briefly, the paraffin‐embedded tissues were sliced prior to H&E staining. Representative areas of the H&E staining sections were evaluated and confirmed by a senior pathologist. A TMA marker was designed by using 1.5 mm tissue core in each case. Finally, TMA containing 16 × 10 tissue cores for all BPH specimens in each were obtained and then sliced continuously into 4‐μm‐thick sections.

### Immunohistochemistry and clinical correlation analysis

The paraffin sections were deparaffinized in xylene prior to anhydrous ethanol, 95% alcohol and 75% alcohol in turn. They were subsequently kept in 10 mM boiled sodium citrate buffer (pH 6.0) for 2 min for antigen retrieval and incubated with 3% H_2_O_2_ solution for 10 min to inactivate endogenous peroxidase. To block non-specific binding, 15% normal goat serum was used to incubate sections for 15 min at room temperature. Next, the sections were incubated successively with GRP78 primary antibody (Table S7) at humidified and 4 °C conditions and with secondary antibody (Table S8) at 37 °C until peroxidase and 3, 3′-diaminobenzidine tetrahydrochloride visualization. Negative controls were incubated with PBS instead of the antibody. All stained sections were imaged using an Olympus-DP72 light microscope (Olympus, Japan). GRP78 expression in the prostate tissues from our TMA was blindly quantified by two pathologists via analysis for positive area of all images with Image J. For further correlation analysis, the clinical data were collected as we mentioned above. Pearson correlation analysis was conducted to investigate the correlation between GRP78 and several clinical characteristics of BPH, as well as GRP78 and multiple markers for apoptosis, EMT and OS.

### Statistical analysis

In this study, all data were expressed in the form of mean ± standard deviation (SD). Statistical analysis was performed by Student’s t-test or one-way ANOVA with SPSS v25.0. *p* < 0.05 was considered as statistically significant.

## Supplementary information

supplementary materials

checklist

author-contribution form

## Data Availability

The data used to support the findings of this study are available from the corresponding author upon reasonable request.
