# Supplementary material for: Glucose-regulated protein 78 modulates cell growth, epithelial–mesenchymal transition, and oxidative stress in the hyperplastic prostate
Source: Cell Death Dis. 2022 Jan 24;13(1):78. doi: 10.1038/s41419-022-04522-4 (PMC8786955; doi:10.1038/s41419-022-04522-4)
Supplement: Supplementary file 1 — supplementary materials [file 41419_2022_4522_MOESM1_ESM.docx]

**Supplementary materials**

**Supplementary figures**

**
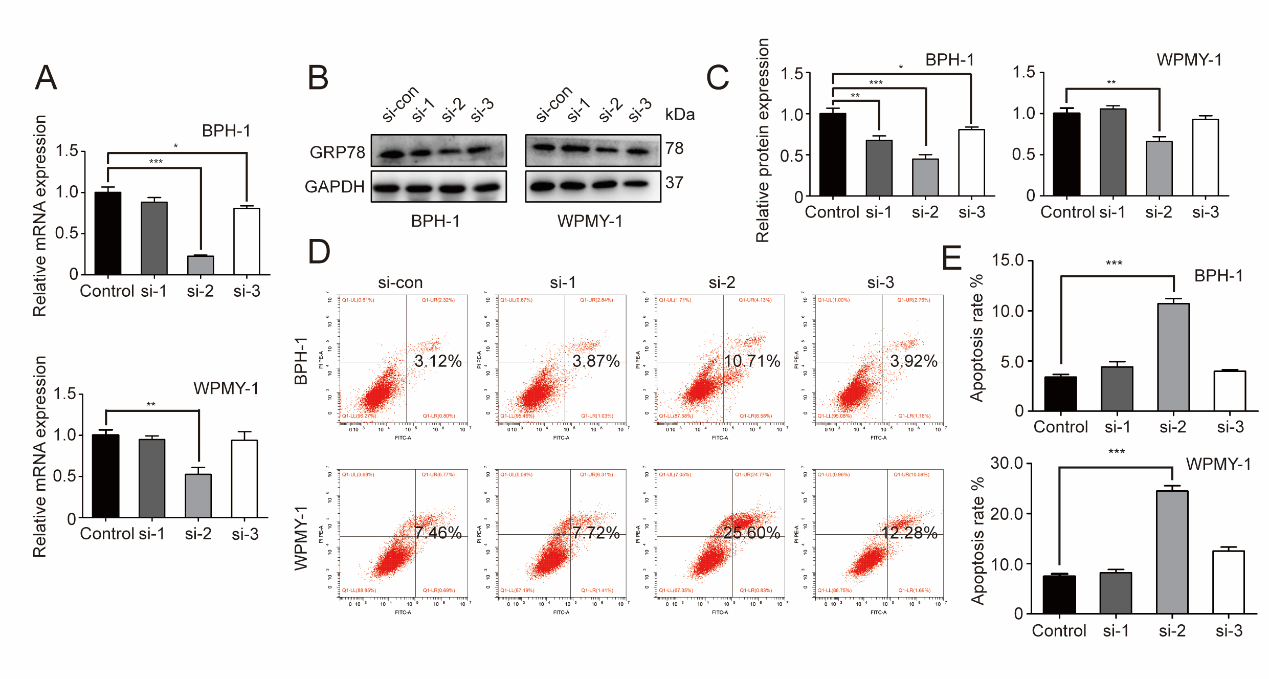
**

**Fig. S1 Knockdown efficiency validation for three GRP78 siRNAs in prostate cells.**

**A:** Determination of knockdown efficiency of three GRP78 siRNAs with different sequences (si-1, si-2 and si-3) at the mRNA levels in BPH-1 and WPMY-1 cells by qPCR. **B, C:** Immunoblot assay and relative densitometric quantification for GRP78 in prostate cells after transfection with si-1, si-2 and si-3, respectively. **D, E:** Flow cytometry analysis for apoptosis of prostate cells transfected with si-1, si-2 or si-3. The apoptosis rate was statistically analyzed. GAPDH is used as loading control. ^*^: *p* < 0.05; ^**^: *p* < 0.01; ^***^: *p* < 0.001.


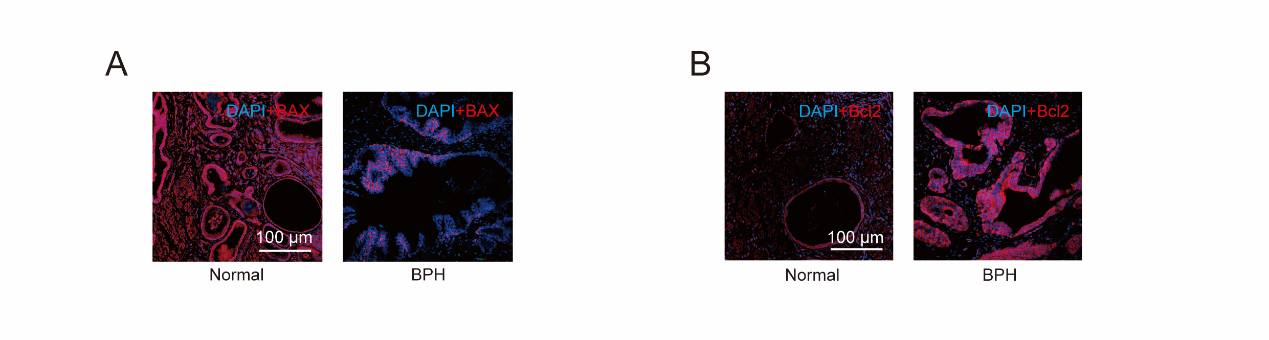


**Fig. S2 The differential expression of BAX and Bcl2 in prostate tissues.**

**A, B:** Immunofluorescence staining for BAX and Bcl2 in the hyperplastic prostate and normal prostate. DAPI (blue) indicates the nucleus staining. Cy3-immunofluorescence (red) represents BAX or Bcl2 protein staining.

**
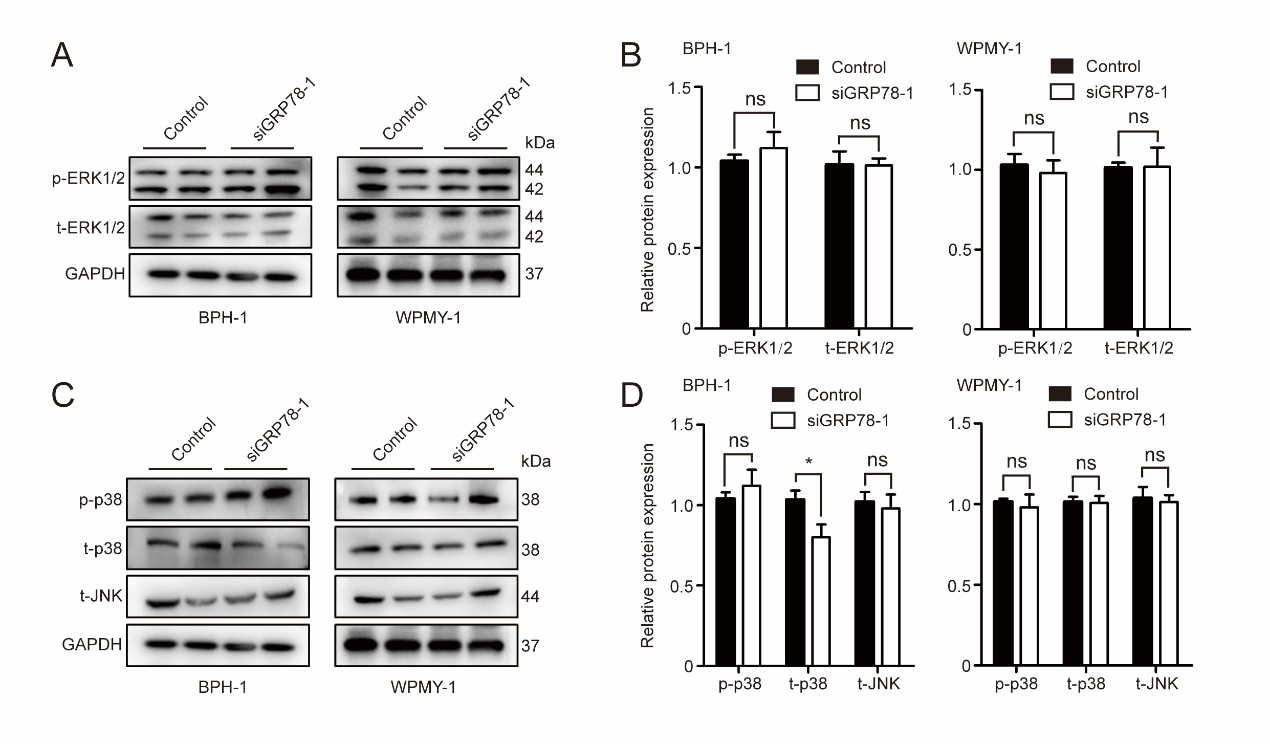
**

**Fig. S3 GRP78 is not the regulator of MAPK/ERK signal pathway in prostate cells.**

**A, B:** The protein expression of molecular markers for MAPK/ERK signaling: p-ERK 1/2 and t-ERK 1/2 in GRP78-silenced BPH-1 and WPMY-1 cells. The relative densitometric quantification of p-ERK 1/2 and t-ERK 1/2 was analyzed. **C, D:** Immunoblot assay and relative densitometric quantification for other markers of MAPK signaling (t-p38, p-p38 and JNK) in GRP78-silenced prostate cells. GAPDH is used as loading control. ns: *p* > 0.05.

**Tables**

Table S1 ROS levels in GRP78 silenced /overexpressed prostate cells

| Cell types | Mean ROS FITC-A | |
| --- | --- | --- |
|  | Control | siGRP78-1 |
| BPH-1 | 182314.3 | 264531.5 |
| WPMY-1 | 102947.6 | 136436.2 |
| RWPE-1 | 172978.9 | 233703.8 |

| Cell types | Mean ROS FITC-A | |
| --- | --- | --- |
|  | Control | siGRP78-2 |
| BPH-1 | 264297.2 | 300325.3 |
| WPMY-1 | 85981.8 | 109183.5 |
| RWPE-1 | 176068.8 | 200069.6 |

| Cell types | Mean ROS FITC-A | |
| --- | --- | --- |
|  | Vector | GRP78-overexpression |
| BPH-1 | 318348.1 | 255817.6 |
| WPMY-1 | 111555.1 | 75655.9 |
| RWPE-1 | 227718.5 | 160061.3 |

Table S2 ROS levels in prostate cells with/without GRP78 knockdown

and SC79 treatment

| Cell types | Mean ROS FITC-A | | | | | |
| --- | --- | --- | --- | --- | --- | --- |
|  | Control | | siGRP78-1 | siGRP78-1+SC79 | Control+SC79 | |
| BPH-1 | 179609.7 | 248219.1 | | 209083.9 | | 172206.5 |
| WPMY-1 | 111528.6 | 164840.0 | | 129089.2 | | 109994.2 |

Table S3 The analysis for clinical correlation between GRP78 and BPH

| Parameters for clinical traits  of BPH | GRP78 | |
| --- | --- | --- |
|  | Pearson Correlation | P value |
| Age | -.146 | .159 |
| BMI | .006 | .958 |
| Prostate volume | -.042 | .686 |
| fPSA | -.035 | .749 |
| tPSA | -.072 | .508 |
| fPSA/tPSA | -.018 | .871 |
| Nocturia | -.417^**^ | .008^**^ |
| IPSS | -.272 | .153 |
| Qmax | .186 | .279 |
| Residual urine | -.190 | .353 |

BMI, body mass index. fPSA, free prostate specific antigen. tPSA, total prostate specific antigen. IPSS, international prostate symptom score. Qmax, maximum urine flow rate. ^**^ *p* < 0.01 (2-tailed)

Table S4 The analysis for correlation between GRP78 and multiple proteins

| *r* value | GRP78 | BAX | SOD2 | Bcl2 | CAT | E-cad | N-cad |
| --- | --- | --- | --- | --- | --- | --- | --- |
| GRP78 | 1 | -0.968^**^ | 0.548^**^ | 0.371^**^ | 0.760^**^ | 0.424^**^ | 0.732^**^ |
| BAX | -0.968^**^ | 1 | -0.537^**^ | -0.357^**^ | -0.760^**^ | -0.404^**^ | -0.726^**^ |
| SOD2 | 0.548^**^ | -0.537^**^ | 1 | 0.218^*^ | 0.533^**^ | 0.323^**^ | 0.687^**^ |
| Bcl2 | 0.371^**^ | -0.357^**^ | 0.218^*^ | 1 | 0.329^**^ | 0.866^**^ | 0.356^**^ |
| CAT | 0.760^**^ | -0.760^**^ | 0.533^**^ | 0.329^**^ | 1 | 0.371^**^ | 0.848^**^ |
| E-cad | 0.424^**^ | -0.404^**^ | 0.323^**^ | 0.866^**^ | 0.371^**^ | 1 | 0.406^**^ |
| N-cad | 0.732^**^ | -0.726^**^ | 0.687^**^ | 0.356^**^ | 0.848^**^ | 0.406^**^ | 1 |

^*^: *p* < 0.05; ^**^: *p* < 0.01

Table S5 Sequences for GRP78 siRNA

| Gene (Human) | Forward primer (5’~3’) | Reverse primer (5’~3’) |
| --- | --- | --- |
| si-GRP78-1 | GGGCAAAGAUGUCAGGAAATT | UUUCCUGACAUCUUUGCCCTT |
| si-GRP78-2 | GAGGCUUAUUUGGGAAAGATT | UCUUUCCCAAAUAAGCCUCTT |
| si-GRP78-3 | GAGGUGUCAUGACCAAACUTT | AGUUUGGUCAUGACACCUCTT |

Table S6 Primer sequences for qRT-PCR

| Gene (Human) | Forward primer (5’~3’) | Reverse primer (5’~3’) |
| --- | --- | --- |
| GRP78 | ACTTGGGGACCACCTATTCCT | ATCGCCAATCAGACGCTCC |
| E-cad | CGAGAGCTACACGTTCACGG | GGGTGTCGAGGGAAAAATAGG |
| N-cad | TCAGGCGTCTGTAGAGGCTT | ATGCACATCCTTCGATAAGACTG |
| vimentin | GACGCCATCAACACCGAGTT | CTTTGTCGTTGGTTAGCTGGT |
| Snail1 | TGCGTCTGCGGAACCTG | GGACTCTTGGTGCTTGTGGA |
| Snail2 | ATATTCGGACCCACACATTACC | ACATTCTGGAGAAGGTTTTGGA |
| Twist | GCCTAGAGTTGCCGACTTATG | TGCGTTTCCTGTTAAGGTAGC |
| ZEB1 | GCTGTTTCAAGATGTTTCCTTCCA | TTACACCCAGACTGCGTCAC |
| ZEB2 | CGCTTGACATCACTGAAGGA | CTTGCCACACTCTGTGCATT |
| SOD2 | TGTGGCCGATGTGT | GCGTTTCCTGTCTTTGTACTTTC |
| CAT | CAGATAGCCTTCGACCCAAG | GTAGGGACAGTTCACAGGTATATG |
| GAPDH | ATGGAGAAGGCTGGGGCTC | AAGTTGTCATGGATGACCTTG |

Table S7 Primary antibodies for Western Blot and immunofluorescence

| Antigens (Human) | Species antibodies raised in | Dilution | Supplier |
| --- | --- | --- | --- |
| GRP78 | Rabbit, polyclonal | 1:1000 (WB)  1:200 (IF)  1:200 (IHC) | Abclonal A0241 |
| AKT | Rabbit, monoclonal | 1:1000 (WB) | Abclonal A17909 |
| p-AKT | Rabbit, monoclonal | 1:1000 (WB) | Abclonal AP0637 |
| mTOR | Rabbit, polyclonal | 1:1000 (WB) | Abclonal A11354 |
| p-mTOR | Rabbit, monoclonal | 1:1000 (WB) | Abclonal AP0115 |
| BAX | Rabbit, monoclonal | 1:1000 (WB)  1:200 (IF)  1:200 (IHC) | Abclonal A19684 |
| Bcl2 | Rabbit, monoclonal | 1:1000 (WB)  1:200 (IF)  1:200 (IHC) | Abclonal A19693 |
| SOD2 | Rabbit, polyclonal | 1:1000 (WB) | Abclonal A1340 |
| SOD2 | Rabbit, monoclonal | 1:1000 (IHC) | Cell Signaling Technology D3X8F |
| CAT | Rabbit, polyclonal | 1:1000 (WB) | Abclonal A18018 |
| CAT | Rabbit, monoclonal | 1:400 (IHC) | Cell Signaling Technology D4P7B |
| E-cad | Rabbit, polyclonal | 1:1000 (WB)  1:200 (IHC) | Abclonal A3044 |
| N-cad | Rabbit, polyclonal | 1:1000 (WB) | Abclonal A3045 |
| N-cad | Rabbit, monoclonal | 1:200 (IHC) | Abclonal A19083 |
| vimentin | Rabbit, polyclonal | 1:1000 (WB) | Abclonal A11952 |
| Snail-1 | Rabbit, polyclonal | 1:1000 (WB) | Abclonal A5243 |
| Snail-2 | Rabbit, polyclonal | 1:1000 (WB) | Abclonal A1057 |
| Twist | Rabbit, polyclonal | 1:1000 (WB) | Abclonal A15596 |
| ZEB1 | Rabbit, polyclonal | 1:1000 (WB) | Abclonal A1500 |
| ZEB2 | Rabbit, polyclonal | 1:1000 (WB) | Abclonal A5705 |
| GAPDH | Rabbit, monoclonal | 1:1000 (WB) | Abclonal AC027 |

Table S8 Secondary antibodies for Western Blot and counterstaining of nuclei

| Secondary detection system used | Species antibodies raised in | Dilution | Supplier |
| --- | --- | --- | --- |
| Anti-Mouse-IgG (H+L)-HRP | Goat | 1:10000 (WB) | Sungene Biotech, China, Cat. #LK2003 |
| Anti-Rabbit-IgG (H+L)-HRP | Goat | 1:10000 (WB) | Sungene Biotech, China, Cat. #LK2001 |
| Anti-rabbit IgG (H+L), F (ab')2 fragment (Alexa Fluor® 488 Conjugate) | Goat | 1:500 (IF) | Cell Signaling Technology, USA, cat. no. 4412 |
| Hoechst 33342 (1 mg/ml) nucleic acid staining (DAPI) | - | 1:750 (IF) | Molecular Probes/Invitrogen, Carlsbad, CA, USA, cat. no. A11007 |
