# Supplementary material for: Glucose-regulated protein 78 modulates cell growth, epithelial–mesenchymal transition, and oxidative stress in the hyperplastic prostate
Source: Cell Death Dis. 2022 Jan 24;13(1):78. doi: 10.1038/s41419-022-04522-4 (PMC8786955; doi:10.1038/s41419-022-04522-4)
Supplement: Supplementary file 3 — author-contribution form [file 41419_2022_4522_MOESM3_ESM.pdf]

DECLARATION OF CONTRIBUTIONS TO ARTICLE

ADMC

Manuscript Number: CDDIS-21-2778RR

Journal Name: *Cell Death & Disease* (the 'Journal')

Proposed Title of the Contribution:

Glucose-regulated Protein 78 Modulates Cell Growth, Epithelial-Mesenchymal Transition, and Oxidative Stress in the Hyperplastic Prostate (the 'Contribution)

Author(s):

Xun Fu, Jianmin Liu, Daoquan Liu, Yongying Zhou, Yuhang Guo, Zhen Wang, Shu Yang, Weixiang He, Ping Chen, Xinghuan Wang, Michael E DiSanto and Xinhua Zhang (the 'Authors')

For all *CDDis* articles, each person named as an author in the published version must be able to show he or she has contributed substantially to the article.

Authorship credit should be based on 1) substantial contributions to conception and design, acquisition of data, or analysis and interpretation of data; 2) drafting the article or revising it critically for important intellectual content; and 3) final approval of the version to be published. Authors should meet conditions 1, 2 and 3.

Any person who cannot be shown to have made a substantial contribution to the article cannot be listed as an author in the final version. The name of any person who is deemed to have made a minor contribution can, however, appear in the Acknowledgments section of the article.

Please complete the table below to indicate the contributions of all named authors to the manuscript.

Author Full Name:	Specification of Contribution to the Manuscript:
Xun Fu	Conception and design, drafting the article, final approval of the version to be published
Jianmin Liu	Acquisition of data,final approval of the version to be published
Daoquan Liu	Conception and design, acquisition of data, final approval of the version to be published
Yongying Zhou	Analysis of data,final approval of the version to be published
Yuhang Guo	Acquisition of data,final approval of the version to be published
Zhen Wang	Analysis of data,final approval of the version to be published
Shu Yang	Analysis of data,final approval of the version to be published
Weixiang He	Analysis of data,final approval of the version to be published
Ping Chen	Analysis of data,final approval of the version to be published
Xinghuan Wang	Analysis of data,final approval of the version to be published
Michael E DiSanto	Revising the article,final approval of the version to be published
Xinhua Zhang	Conception and design, revising the article, final approval of the version to be published

Please complete the table below to indicate the contributions of all named authors to the figures.

Figure 1:

Xun Fu, Jianmin Liu, Ping Chen, Yongying Zhou, Weixiang He, Xinhua Zhang

Figure 2:

Xun Fu, Jianmin Liu, Yuhang Guo, Daoquan Liu, Xinhua Zhang

Figure 3:

Xun Fu, Yuhang Guo, Daoquan Liu, Ping Chen, Shu Yang, Xinhua Zhang

Figure 4:

Yongying Zhou, Yuhang Guo, Daoquan Liu, Weixiang He, Zhen Wang, Xinhua Zhang

Figure 5:

Jianmin Liu, Xun Fu, Yongying Zhou, Xinghuan Wang, Michael E DiSanto, Xinhua Zhang

Figure 6:

Xun Fu, Jianmin Liu, Zhen Wang, Shu Yang, Xinghuan Wang, Xinhua Zhang

Figure 7
Xun Fu, Yuhang Guo, Yongying Zhou, Michael E DiSanto, Xinhua Zhang

Figure 8
Xun Fu, Jianmin Liu, Ping Chen, Daoquan Liu, Zhen Wang, Xinhua Zhang

Signed for and on behalf of the Author(s):	Print Name:	Date:
	Xinhua Zhang	14/12/2021
